# Researches, Historical, Topographical and Critical, on Yellow Fever

**Published:** 1846-12

**Authors:** 


### ARTICLE X.

*Researches, Historical, Topographical and Critical, on Yellow Fever.* By BENNETT DOWLER, M.D., of New Orleans.

Dr. Dowler is one of the most racy writers in this country,—is a close observer, diligent and thorough in his investigations, and constantly on the look out for something new. We always expect a treat when we find his name as author at the head of an article. The quaintness of his style often serves to spice up what might otherwise be considered the dry detail of facts.

The aim of the present article appears to be, to prove, that while modern, the yellow fever is neither contagious or miasmatic, exclusively Asiatic or European in its origin. The author makes numerous references to prove that had yellow fever prevailed previous to the discovery of America, some of the numerous authors would have mentioned it, and quotes Mr. Webster on pestilence, to prove that the disease prevailed amongst the aborigines of our country, prior to the European settlements.

He deals out blows against malaria and contagion with a masterly hand as follows:

"Barbadoes was settled by the English in 1605, and was soon put in a high state of cultivation. In less than a century, it contained about 150,000 inhabitants, more than 500 to the square mile—a density exceeding that of nearly all other countries. In 1786, the population had declined to less than 80,000—a result, perhaps, more attributable to the exhaustion of the soil, than to the ravages of yellow fever: for this island like most places to which yellow fever shows a geographical predilection, is, paradoxical as it may seem, extremely healthful, being little, if at all subject to other forms of epidemic fever. Lind, repeatedly calls it the most 'pleasant and healthful' of the West Indian Islands. Towards the close of the eighteenth century, an English traveller, Sir W. Young, thus describes it: 'The Island is dotted with houses as thickly as on the declivities in the neighborhood of London or Bristol, but with no woods; two or three straggling cocoas near each dwelling, were all the trees to be seen.'

"For two centuries, this island has been the hot-bed of yellow fever, which Dr. Ferguson, Inspector General of hospitals in the West Indies, declares to be of the very worst form.* If the logic of contagionists is inconclusive, that of miasmatists is evidently erroneous. No one can deny that temperature, humidity, and the like, greatly influence human health. It does not hence follow that the swamps of Louisiana emit any mystical miasma, (any more than so much rose water,) which is the cause of the yellow fever: indeed the most swampy portions of the state are freest from this disease. When swamps cannot be found, as at Barbadoes, Vera Cruz, Havanna, not to name scores of towns in Spain, including that dry mountain of rock, Gibraltar, still the advocates of this doctrine would have us to believe, in opposition to the evidence of our senses, that as the disease is present so is its assumed cause, marsh exhalation. This extensive subject can only be glanced at in this place. Bancroft † attempts to account for the great epidemic of 1647, not by the agency of swamps, but by stating, that, as, Barbadoes had been then settled but little more than twenty years, and, 'that so little of it was at that time cleared and cultivated, that *dry* weather assisted by great heat, was best suited to the production of noxious miasma; contrary to what has been the case at Barbadoes since it attained its highest state of cultivation many years ago.'—He totally forgets, that as the cultivation increased, so did the yellow fever for two centuries.

"It is worthy of observation, that intermittents are not found in this island. Dr. Gilpin, principal medical officer at Gibraltar, never knew of but one intermittent in that great fortress of yellow fever; yet, miasmatists persist in referring these two maladies to the same cause. This, however, is a subject which will be investigated elsewhere, and is merely alluded to here, in connection with the topography of the island under consideration.

"Sufficient number of authorities exist, to prove that a great yellow fever epidemic prevailed in this island, in the year 1647, already mentioned. These authors having been contemporaneous with each other, with the events which they have recorded, agreeing in their relation of the same, and having been without any interested motive to deceive themselves or others, their testimony, as far as it goes, is entitled to the utmost credit. Their history of symptoms is necessa-

* Med. Chir. Trans. † On Yel. Fev.

rily imperfect, representing in this respect, the science of medicine at that dark period. Dr. R. Vines, a planter and physician, describes this epidemic at Barbadoes, as 'a plague very infectious, first attacking the ablest men of the greatest bodily ability.' Ligon, whose history of Barbadoes appeared ten years after the epidemic, arrived in that island early in September. He says, that before the close of that month the living were hardly able to bury the dead.* He gives a very remarkable trait of yellow fever, which two centuries have confirmed, and one which is, in the histories of maladies, very peculiar; that '*for one woman that died, there were ten men.*" The mortality was five or six thousand in this small and infant colony!

"Father Du Tertre, who lived in the West Indies, in 1635, wrote an account of an epidemic, which prevailed in St. Christopher, before that already mentioned in Barbadoes, and which he called '*la peste*,' and described as being accompanied with headache, constant vomiting, and death in three days. It destroyed, in three months, one-third of the entire population of that island.

"The assumption, that yellow fever is the product of marsh poison, is not only unsupported by its topographical affinities but is irreconcileable to its modern appearance

"Sterne has somewhere asserted, that our earth is the vilest and dirtiest, and, therefore, the most miasmatic, it may be supposed of all the planets, being made wholly out of the refuse clippings of the rest. The geological account of its primary condition, is not very flattering in a sanitory point of view; since upon the theory of marsh exhalation, yellow fever must have been as old as creation; and withal, infinitely more common and fatal in ancient than in modern times. The land as it arose from the water, boggy, fenny and marshy, shot forth gigantic ferns, and rank herbage of many kinds, which, judging from their fossil remains, now constituting the immense coal fields of our globe, were more like our present trees, in luxuriance, than the aquatic plants of our era. Mountains upheaved by volcanic action, by enclosing vast basins, into which alluvial matter was constantly descending, for countless ages, formed vast swampy areas, from which a concentrated malaria, equaling that which probably followed the Noahic flood, must have caused yellow fever epidemics, sufficient to have destroyed, at least, the males of the Caucasian race, or a great proportion of them, though many white females, and nearly all the negroes, might have escaped then, as now.

"To say nothing of the valley of the Mississippi, its delta alone, from Cape Girardeau to its mouth forms, according to Mr. Forshey, 21,200 square miles, having a mean width of

* Bancroft and Webster.

418 miles, and a length of 600; the depth of the alluvion having a mean of 50 feet, required 13,684 years for its formation, by deposits from the river.* During this long period it must have been excessively swampy. The condition in which De Soto found it, in 1539, was of course worse than at present;† yet, the yellow fever did not appear in New Orleans until a period very recent, when compared with the Northern States and the West Indies, which had been previously desolated by many epidemics, for nearly a century and a half before it visited swampy Louisiana. The valley of the Mississippi, not the little fens of Old England, nor the mill-ponds of New England, nor the Pontine marshes, or rather the Pontine pasture lands of the Eternal city, offers the best and most extensive field for testing every possible malarial question which the most imaginative miasmatist has ever been able to conceive or propound since 1717, when Lancisi published his *De Noxiis Paludum Effluviis.* In the entire valley, an erea of nearly the third of a million of square miles, since its discovery, the aggregate mortality from yellow fever to the present time, has not equalled the half of that during a single year in Old Spain, where it has amounted by the official reports to one hundred and twenty thousand, in places free from swamps, and even among mountains.

"Omitting the palpable anachronism of the alledged Asian importation, there seems to be as little plausibility as probability, in seeking the cause of a malady, in a country where the malady itself, is unknown. It may be patriotic, however, to defend our country from imputed contagion, right or wrong, and to throw the blame of yellow fever upon our antipodes, though without better evidence than has yet been adduced, this process is far from being altogether scientific. Charge the Siamese with originating the contagion—they will charge the Japanese or some other people. Thus contagion, like the Wandering Jew, will be driven "from Indus to the Pole," and from the pole to the burning zone. March! March! will be the universal cry. It is affirmed, poetically no doubt, that the great pendulum of the clock of Eternity in its vibrations utters but two words, EVER! NEVER! So it is with this endless question of contagion—*Ever* assuming, *Never* proving its conclusions. Doomed to march! march! it crosses the Atlantic, doubles the Cape of Good Hope, traverses the Indian Ocean, passes through the Straits of Malacca—a distance of twenty thousand miles, to get a product, which a crowd of facts

* Newspapers, 1846.

† So little has the Louisiana coast, along the Gulf, changed, since 1685, when La Salle committed the fatal mistake of landing—not at the mouth of the Mississippi, as he intended—but west of it, that Mr. Darby has been able to identify the exact place of his debarkation, between the Vermillion and the Mermentau rivers, from the minute description, at that period, of this shore, with its extremely uniform, low, shining banks of sand; all of which, he declares, he took some pains to ascertain.—*Descrip. La.*, 1817.

show to be indigenous to certain places of the western hemisphere, or to certain local climates, acting during the hot season, and for only a few years, on unassimilated constitutions not long resident in cities or villages.

"The cause, which in one locality, produces gôitre; in another, elephantiasis; in a third, Plica Polonica; in a fourth, the yaws; in a fifth, leprosy; in the sixth, yellow fever—is quite unknown, though many pretended explanations have been given. As some places produce wheat, or bananas; live oaks or cypresses; hanging moss or palmettos; mosquitoes or alligators—so may yellow fever be produced, without our being able to show the cause of the one or the other class of effects.

"That the essential cause of yellow fever will ever be discovered, or being discovered, will be controlled or prevented by human art is altogether improbable. Its mysterious cycles culminate, decline, and re-appear. Charleston, desolated at the close of the seventeenth century, was exempt in the first quarter, but a sufferer in the second quarter of the eighteenth —nearly half a century of exemption followed again—(a period much longer than that which now cheers the cities of New York, Philadelphia, Boston, and Baltimore, with the hope that the yellow fever has taken its leave of them forever.) But the last decennial period of the past century, and the first of the present, relumed the flames of the epidemic in Charleston where they had smouldered so long, and in which they continue to break out almost annually. Charleston suffered nearly a century in advance of New Orleans, and is still as great a sufferer as the latter.

"It is to be lamented that the topographical ameliorations which have been pushed forward with a celerity characteristic of New Orleans, have not diminished or modified the yellow fever of the place, though strange to say, this opinion is so unwelcome to all, and so humiliating to theorists, that it is constantly repudiated, while 'the long funerals which blackened' all the streets, no less than five different seasons, from 1837 to 1843, inclusive, are still but too fresh in the recollections of a hundred thousand people. As gloomy prognostications are as useless as unwarrantable, let us hope that New Orleans has entered upon a non-epidemic cycle, not only for half a century, as once happened to Charleston, but forever. The yellow fever prophets and prophetesses, have not, hitherto, been able to read the Sibylline leaves of its etiology, so that epidemics can be certainly known until after their occurrence.

"The rejection of one error never justifies the adoption of another, unless it be on the principle of Dean Swift, that all happiness consists in being *well* deceived, or on that of Fontanelle, which makes philosophy itself consist in much curiosity and very bad eyes. To explain the cause of yellow fever seems to be regarded, not as supererogation, but as a paramount duty enjoined in the medical decalogue. It is scarcely reckoned a sin against logic, to resort to almost any etiological *non-sequitur*, for this purpose. The cause is assumed; when the effect appears without the cause, either nothing is said of the absence, or some other cause, condition, or circumstance is affirmed as a sufficient substitute for the truant; when the assumed cause exists in the greatest concentration, without the presence of any effect whatever, some assumed contingency is supposed to counteract its power: as a *ruse de guerre*, a mere incident, will, however, always answer, and can always be found:—dry or wet; hilly or marshy; rocks or mud; vegetable or animal matter; stagnant air or storms; heat or cold; infected ships, or a 'pair of trowsers;' immigrants or insects; gases or graveyards; absolute or conditional contagion or malaria, both foreign or domestic. This sort of ratiocination was not altogether unknown to Joe Miller:—'The Frenchman who observed that an Englishman recovered from a fever after eating a red herring, administered one to the first of his fellow countrymen whom he found laboring under that disease, and having found that it killed him, noted in his tablet that a red herring cured an Englishman of a fever, but kills a Frenchman.' 'Rotten coffee' is found in Philadelphia; three barrels of spoiled mackerel, sour-crout, sour porter, and rotten corn, two thousand pounds of bad bacon, with the heads and entrails of some catfish, are found in a certain town in the State of Mississippi; and a 'trash wharf' in the city of Augusta, Georgia, etc.—these are gravely substituted for contagion, for 'a dirty pair of trowsers,' (from Martinico,) in which, according to the most 'potent' authorities of a New England city, a most deadly epidemic was imported. Have not 'dead fish,' 'trash,' 'dirty trowsers,' and the like, abounded since the foundation of the world, in all climates and places? Does not the law of continuity in the cause, ['trash,'] require a corresponding continuity in the effect, ['yellow fever?'] Were yellow fever producible by a few pounds of 'rotten coffee,' would not the incendiary, when sated with conflagrations, amuse himself, by way of variety, by kindling up an occasional epidemic, especially if he were himself acclimated, and wished to profit by the commercial speculations always incidental to such an event, whether his stock in trade consists of cotton, sugar, red herrings, drugs or coffins.

"In denying, or rather doubting, that yellow fever is produced by any emanation from the sick, or from marshes, nothing more is intended than such emanation is unsupported by any evidence worthy of belief. Were a subterranean, a solar, a lunar, or a stellar theory substituted, it might be true or not; the *onus probandi* belongs to the proposer of such doctrines.

"An alledged cause ought to be invariably followed by its effects: for example, if the doctrine of imported contagion be adopted, then the town of the Balize, at the mouth of the Mississippi, is the most exposed point on the globe, as the vessels supposed to be infected, are often detained there for want of pilotage, and towage, and from getting fast on the bar; adopt miasma as the cause, and yellow fever ought to be eternal, as this is, of all towns, the most exposed to marsh exhalation, and yet yellow fever is unknown to the residents.

"The *morale* of contagion and miasma is very dissimilar: the one is anti-social, repulsive, dooming its victims to cheerless insulation—to withering neglect. It cries—stand off!—perish in ships!—perish in lazarettos!—perish in hovels!—'let the dead bury the dead!' The other, like the good Samaritan, fearing nothing from contagion, offers not only personal attendance but sympathy in the hour of need. Besides the utilitarianism of this latter drectrine stands forth, actualized in the improving, draining, cleansing and embellishing of both town and country localities. Yet in the sciences, the utility of an error cannot plead its justification. Sciolism may declaim against the pulling down of existing systems. It seems to abhor a vacuum. But the massive columns of truth will never arise until the foundation shall be clear." E.

